# Tick‐Tac‐Foe: When Ticks, Trade, and Zoonotic Pathogens Align in African Wet Meat Markets

**DOI:** 10.1002/puh2.70261

**Published:** 2026-05-01

**Authors:** Allen Takudzwa Munaro

**Affiliations:** ^1^ Department of Biology School of Natural Sciences and Mathematics Chinhoyi University of Technology Chinhoyi Zimbabwe

**Keywords:** live markets, One Health, tick‐borne pathogens, ticks, wet markets, zoonosis

## Abstract

Zoonotic diseases account for over ∼60% of infectious diseases and present a significantly growing fatality threat in Africa. Live and wet markets (LWMs) in Africa function as key economic venues that support human livelihoods through social interaction and trade in food stuff, including meat and other animal‐based products. These spaces concentrate and exaggerate human and animal contact, creating conditions conducive to zoonotic spill‐over events. This narrative review, based on an opportunistic literature identification process, summarizes the diversity of ticks and tick‐borne pathogens (TTBPs) associated with carcasses at African LWMs, as well as the awareness, perceptions, and control practices of stakeholders along the meat value chain. Findings show pervasive infestations by *Amblyomma*, *Rhipicephalus*, *Hyalomma*; limited reports of *Haemaphysalis*, *Ixodes*, and *Demacentor* species; along with the circulation of *Rickettsia africae*, Crimean‐Congo hemorrhagic fever virus, *Anaplasma* spp., and *Coxiella burnetii*. Surveillance efforts remain geographically patchy, with strong bias towards East and West Africa. The knowledge of tick‐borne zoonotic diseases is poor and shaped by sociodemographic factors, like education and occupational roles. Control measures, where practiced, are inconsistently applied and inadequate. Collectively, these findings flag African LWMs as understudied epidemiologically permeable hubs, with potential implications in the ecology of tick‐borne zoonotic diseases.

## 1 Introduction

Zoonotic diseases, the ailments capable of transcending the human–animal boundary, represent the majority (60%) of infectious threats worldwide and have become a leading public health concern [1, 2, 3, 4]. Despite their profound nature, increased frequency, and transmissibility [5], public awareness and interest in zoonotic diseases only heightened post the COVID‐19 pandemic [3]. As a result of improved monitoring, 3 billion cases of human ailments and 2.7 million deaths are now attributed to zoonotic diseases every year [4], presenting a significant public health burden. In Africa, zoonotic diseases alone cause an estimated pooled fatality rate of ∼345.4 deaths per 1000 [6]. Thus, research addressing zoonotic risks is key to public health resilience and realizing One Health goals.

Vectors play a significant role in the transmission of zoonotic and infectious diseases [7, 8], accounting for more than 17% of all infectious diseases (World Health Organization 2023). Ticks, the blood‐sucking external parasites of terrestrial and semiaquatic vertebrates, transmit a wide range of bacterial, viral, and protozoan pathogens considered to be zoonotic [9]. Thus, ticks are vectors of epidemiological concern. For instance, *Haemaphysalis longicornis* ticks have been recently shown to transmit severe fever with thrombocytopenia syndrome (SFTS), a deadly tick‐borne disease (TBD) caused by the *Dabi bandavirus* [10]. Because ticks are considered interspecific bridge vectors with a wide host specificity spectrum [11, 12, 13, 14], the risk of zoonoses at epidemiologically permeable hubs can be predicted to be high. This is particularly alarming as reseach has primarily focused on the veterinarian and conservation implications of tick‐borne pathogens (TBPs) in the African context [15, 16], ignoring the epidemiological significance of ticks and TBPs (TTBPs).

In Africa, human livelihoods (e.g., food, income, and medicine) are still heavily reliant on animals [17, 18, 19, 20, 21], facilitating high levels of contact between humans and the surrounding fauna [6, 22]. Thus, the risk of zoonoses is potentially exaggerated in these overcrowded, low‐ and middle‐income countries [22] pervasively lacking basic sanitation facilities and primary healthcare [23, 24]. Inevitably, this dire situation creates a feedback cycle that reinforces dependency on animals, resulting in a complex nexus where business, nature, and health collide, albeit limited public health monitoring. These ecologically permeable economic hubs are locations where frequent human–animal interactions make disease spread more likely. One such hub occurs at live and wet markets (LWMs), where domestic and wildlife carcasses are traded to sustain livelihoods [25].

**FIGURE 1:**
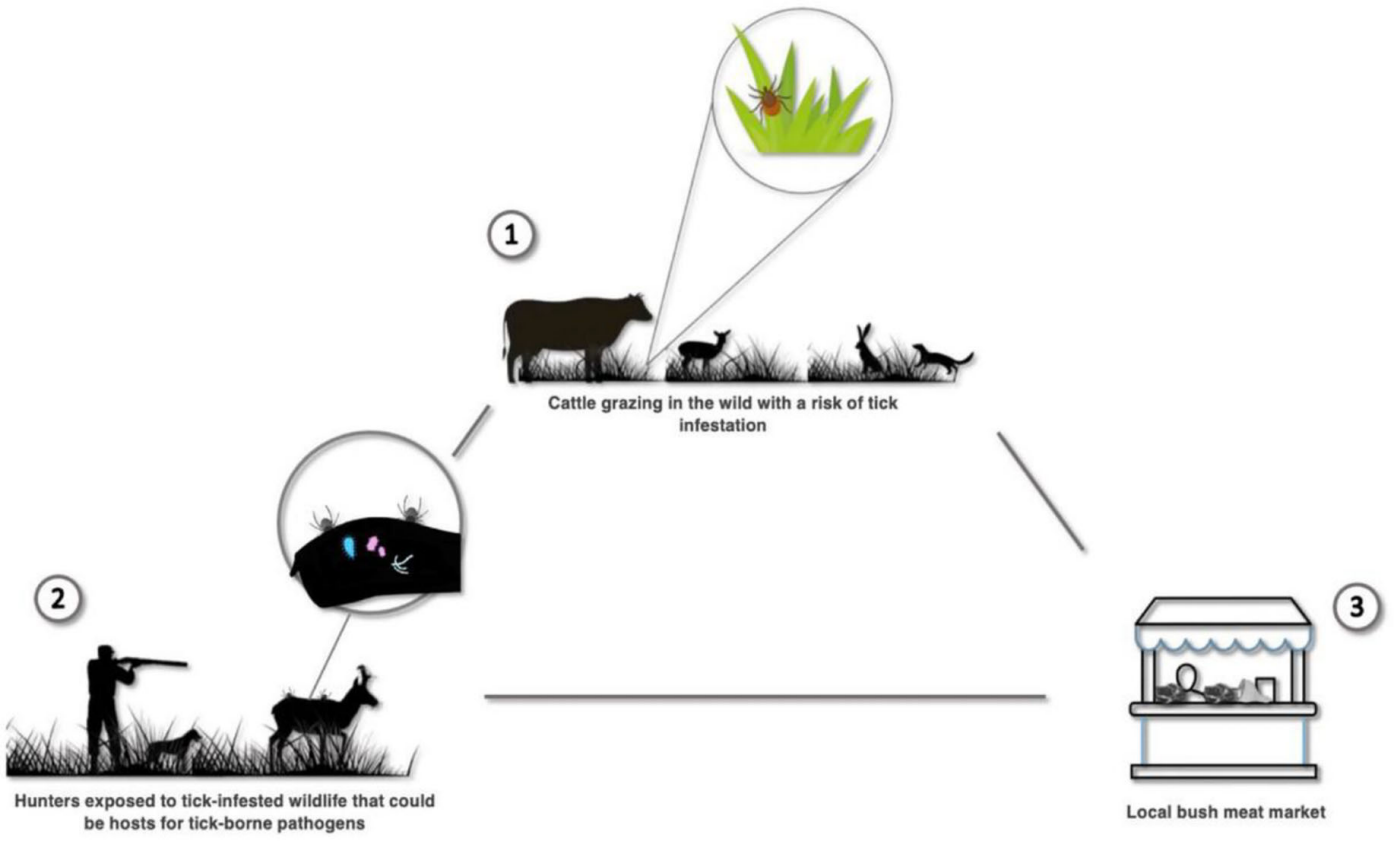
Pathways of human exposure to tick and tick‐borne zoonosis at LWMs (adopted from [28]): (1) grazing exposure—domestic and wild animal risk tick bites and tick‐borne pathogen infection during grazing; (2) processing exposure—hunters, farmers, and slaughterers risk tick bites and zoonotic transmission when killing and handling animals; and (3) market exposure—butchers, traders, and consumers risk tick bites and associated zoonotic transmission during cutting, selling, and buying at LWMs.

LWMs typically refer to widespread, semi‐open, and somewhat regulated formal and informal spaces where animals are often slaughtered and sold on‐site, and fresh products are traded without adequate cold‐chain systems [26]. Commonly, many people access daily supplies, interact, and generate income in these urban economies that vary by country, culture, and dietary preferences [25, 27] In the African context, LWMs equally involve the trade of domestic and wild animals and are deeply rooted in culture than dietary preferences. Such a system exaggerates the TTBP exposure pathways (see Figure 1 for pathways of human exposure to tick and tick‐borne zoonosis at African LWMs). In contrast, the trade in live‐animal products is virtually nonexistent in other regional “wet markets,” such as those found in Europe and America. Comparatively, the African population is at a much higher risk of tick‐borne zoonosis than any other region. Partially, this could explain why zoonotic diseases reportedly result in an estimated pooled fatality rate of ∼345.4 deaths per 1000 in Africa [6].

This narrative review, based on an opportunistic literature identification strategy, synthesizes relevant literature and provides an overview of the current state of knowledge regarding TTBP among informal and semiformal stakeholders in the African meat (domestic/wildlife) trade industry. Interestingly, some African slaughterhouses functionally resembled LWMs due to the deterioration or lack of infrastructure and lack of key utilities, including sanitary electricity for refrigeration [29]. As a result, an attempt was made to consider studies reported across formal and informal abattoirs as they are embedded in the value chain of wet meat markets. The aims of this study were to (a) characterize tick diversity and associated TBPs in these markets and (b) assess the awareness and perceptions of tick‐borne zoonoses among stakeholders. Furthermore, the study aims to (c) evaluate the practices and control measures for TTBPs along the value chain in the African informal meat industry by summarizing hypothetical risk pathways of pathogen transmission from ticks to humans in the African context. To establish a holistic understanding of the knowledge of TTBPs, the knowledge of livestock keepers and hunters was also included in this review.

## 2 What You Can Buy and Sell on African LWMs

Buying and selling meat in Africa is influenced not only by protein needs or aesthetics but also by local availability, profitability, family size, and cultural beliefs. Almost all terrestrial vertebrates are consumed African menus and are therefore traded at LWMs. Domestic animals are the primary protein sources, contributing 30%–80% of agricultural gross domestic product in African countries [30]. Yet harvesting wildlife for meat, medicine, and other products remains common throughout Africa [31]. Thus, a wide spectrum of animals (see Table 1), including reptiles, rodents, ungulates, carnivores, primates, and many bird orders, are hunted and traded at LWMs across Africa [32, 33, 34, 35, 36, 37, 38, 39, 40, 41, 42]. Together, these animals are key tick hosts and reservoirs of tick‐borne zoonotic diseases [42].

**TABLE 1:** Animal carcasses reportedly sold at African live and wet markets (LWMs) reported in this study.

Type of animal	Order	Species list of carcasses sold at African LWMs		Global IUCN red list status	References
		Scientific name	Common name		
Wildlife	Rodentia	*Thryonomys swinderianus*, *Atherurus africanus*	Grasscutter, African brush‐tailed porcupine	LC	Adenyo et al. [38]; Ayodele and Bamidele [43]; Paguem et al. [42]
	Primate	*Cercopithecus nictitans*, *Cercopithecus cephus*, *Cercopithecus pogonias*, ***Cercocebus torquatus***, ***Lophocebus albigena***	Putty‐nosed monkey, moustached monkey, crowned guenon, **red‐capped mangabey**, **grey‐cheeked mangabey**	LC, NT, EN, VU	Linder and Oates [44]; Covey and McGraw [45]
	Artiodactyla	*Philantomba monticola*, *Cephalophus dorsalis*, *Potamochoerus porcus*, *Phacochoerus africanus*	Blue duiker, bay duiker, red river hogs, common warthog, red‐flanked duiker	LC, NT, LC, LC	Paguem et al. [42]
	Pholidota	***Phataginus tricuspis***	**White‐bellied pangolin**	EN	Ayodele and Bamidele [43]; Paguem et al. [42]
	Squamata	*Varanus niloticus*, *Python sebae*	Nile monitor, African rock python	LC, NT	Paguem et al. [42]
	Carnivora	***Caracal aurata***, *Civettictis civetta*	**African golden cat**, African civet	VU, LC	Paguem et al. [42]
	Eulipotyphla	*Atelerix albiventris*	Four‐toed hedgehog	LC	Ayodele and Bamidele [43]; Paguem et al. [42]
	Lagomorpha	*Lepus victoriae*	African savanna hare	LC	Paguem et al. [42]
Domestic	Galliformes	*Numida meleagris*, *Gallu*s spp.	Helmeted guinea fowl, chickens	Unavailable	Bunza et al. [32]
	Columbiformes	*Columba livia*	Domestic pigeon	Unavailable	Bunza et al. [32]
	Artiodactyla	*Bos taurus*, *Capra hircus*, *Ovis aries*, *Sus scrofa domesticus*, *Camelus dromedarius*	Cattle, goats, sheep, pigs, camels, donkeys	Unavailable	Abouelhassan et al. [46]; Chiuya et al. [39]; Ikpeze et al. [33]; Wampande et al. [47]; Yao‐Acapovi et al. [48]; Yousseu et al. [41]
	Perissodactyla	*Equus africanus*, *Equus ferus*	Donkeys, horses	Unavailable	Kamani et al. [36]; Lorusso et al. [49]

*Note:* IUCN statuses: LC—least concern, NT—near threatened, EN—endangered, VU—vulnerable. All animals in bold are endangered or vulnerable.

## 3 Arguments Against LWMs

LWMs have been criticized for their role in facilitating interspecific transmission of infectious and zoonotic pathogens [26, 50]. This is particularly true where carcasses of domestic and wild animals are processed and sold together [42, 47]. Handling, slaughtering, butchering, and eating undercooked and infected meat products exposes actors/stakeholders to zoonotic disease transmission [51, 52]. Moreover, LWMs act as sink points for trans‐boundary movement of vectors and zoonotic pathogens [48, 53]. For instance, the introduction of *Hyalomma impeltatum*, an anthrophilic tick, was reported at a slaughterhouse in Gabon [54]. Such ecologically permeable hubs may have facilitated the spread of the invasive *Rhipicephalus* (*Boophilus*) *microplus* and its associated pathogens, as discussed by Kasaija et al. [55]. In addition to pathogen transmission risks, LWMs also expose humans to hazardous levels of heavy metals [56, 57], generate poor quality effluents [58, 59] capable of spreading pathogens [60, 61], and facilitate trade in wild animals of conservation concern (see Table 1). In Asia, closure of LWMs to curb zoonotic disease transmission has been reported to show between 68%–100% success rate [25, 60, 62]. The adoption of such interventions could prove difficult in Africa due to poverty and lack of employment, among others. Domestication of wildlife is viewed as an alternative and sustainable way, alleviating pressure on wildlife species harvested for consumption [63, 64] and possibly curbing zoonosis from wildlife. However, the success of such efforts is yet to be ascertained.

## 4 TTBPs at African LWMs

All vertebrates, including species traded at African LWMs, serve as hosts for TTBPs. Parasites and pathogens at African LWMs can be organized by genus to highlight differences in host range, ecological behavior, and zoonotic risk. Grouping records into genera such as *Amblyomma* and *Rickettsiae* clarifies patterns of prevalence, host‑switching, and surveillance gaps across regions. This structure also helps pinpoint where targeted monitoring and taxonomic confirmation are most needed to reduce both animal and human health threats.

### 4.1 Ticks Groups

#### 4.1.1 *Amblyomma* Species

*Amblyomma variegatum* is the most widely collected tick, infesting ruminants throughout the reports at various African LWMs and included in this study. Its widespread presence (Walker et al. 2003) likely explains its occurrence in many studies. Infestation rates across slaughterhouse surveys vary from ∼10% to ∼90% from Eastern to Central and Western Africa [37, 39, 41, 47, 48, 65]. Notably, ecological flexibility is common across *Amblyomma* species. For instance, *A. variegatum* can also infest free‐range chickens [66], camels [46], Nile monitors, and four‐toed hedgehogs [42], suggesting generalist feeding behavior and wide host range. Similarly, *A. compressum* was found on seven new wild hosts: African gold cats, antelopes, civets, monkeys, Nile monitors, porcupines, and warthogs [42]. Moreover, *Amblyomma flavomaculatum* ticks were collected for the first time on the carcasses of four wild hosts, including pangolins, hedgehogs, monkeys, and warthogs [42]. These new associations challenge earlier views that these ticks are specialists on pangolins [67] and reptiles [68], suggesting spillover potential, which may increase both the survival and pathogenic potential of these ticks [69]. Other *Amblyomma* ticks reported included *Araneus gemma* [39] and *Abutilon lepidum* [46, 70], adding to the diversity of *Amblyomma* species at LWMs.

#### 4.1.2 *Rhipicephalus* spp

Taxonomically, *Rhipicephalus* ticks represent the most diverse genus collected at African LWMs, with boophilids, *Rhipicephalus decoloratus*, *Rhipicephalus annulatus*, and *R. microplus*, dominating infestations in cattle and small ruminants [33, 39, 41, 47, 48]. Surveillance efforts at slaughterhouses reveal that infestation rates frequently exceed ∼60% [37, 48], underscoring their veterinary and zoonotic significance. Importantly, *R. microplus* has been encountered on nontraditional hosts, such as hedgehogs and Nile monitors [42], keeping in line with earlier reports that it can be found on other domestic and wild hosts [71]. This finding suggests host‐switching that could facilitate pathogen spillover across the wildlife–livestock–human interface. Host switching can amplify the risk of disease transmission from multiple host sources. Other *Rhipicephalus* reported across African LWMs included *Rhipicephalus* (*Boophilus*) *geigyi*, *Rhipicephalus muhsamae*, *Rhipicephalus simus*, *Rhipicephalus linnaei*, *Rhipicephalus guilhoni*, *Rhipicephalus moucheti*, *Rhipicephalus camicasi*, *Rhipicephalus lunutatus*, *Rhipicephalus evertsi*, and *Rhipicephalus pulchellus*, as well as unidentified *Rhipicephalus sanguineus* (s.l.) and *Rhipicephalus* spp. on domestic and wild animals [41, 42, 65, 70]. Collectively, the diversity of *Rhipicephalus* ticks and their host associations could exacerbate zoonotic exposure at LWMs. Indeed, *R. microplus*, *R. guilhoni*, *R. muhsamae*, and *R. linnaei* have been reported to parasitize people [42] and transmit diseases, such as severe fever with thrombocytopenia syndrome virus (SFTSV) [72].

#### 4.1.3 *Hyalomma* spp

*Hyalomma* ticks are among the most widespread external parasites of African animals, with *Hyalomma dromedarii*, *Hyalomma truncatum*, and *Hyalomma rufipes* reported across diverse agroecological zones [70, 73]. Infestation rates vary widely, from ∼2% in some surveys [49] to nearly ∼70% of *H. dromedarii* in camel populations [36, 70], reflecting either true ecological differences or cross‐sectional sampling bias. Importantly, a wide spectrum of species, including *H. dromedarii*, *Hyalomma excavatum*, *Hyalomma rufipes*, *Hyalomma impeltatum*, *Hyalomma marginatum*, *Hyalomma marmoreum*, *Hyalomma lusitanicum*, *H. truncatum*, *Hyalomma nitidum*, *Hyalomma impressum*, *Hyalomma albiparmatum*, *Hyalomma scupense*, and *Hyalomma turanicum*, have been encountered on domestic and wild animal carcasses across African LWMs [42, 48, 49, 53]. However, the bulk of literature relies on convenience and cross‐sectional sampling, with limited molecular tick identification (e.g., 48, 53]), reducing confidence in the true burden. Elsewhere, reports of *H. truncatum* and *H. rufipes* on nontraditional hosts such as hedgehogs and Nile monitors [42] suggest ecological plasticity. Such host switching may increase opportunities for pathogen spillover at LWMs, such as those present at LWMs. The introduction of *H. impeltatum* into Gabon via cattle trade [54] implicates anthropogenic influences as drivers of tick dispersal. Parallel to this, Horton et al. [53] reported that livestock imported from Ethiopia and Somalia carry ticks to Djibouti. Yet systematic surveillance of *Hyalomma* ticks remains patchy at LWMs, particularly in southern and northern Africa, and longitudinal studies are virtually nonexistent.

#### 4.1.4 Underreported Tick Genera

Despite more sporadic reports than *Amblyomma*, *Rhipicephalus*, and *Hyalomma*, other tick genera at African LWMs may play underestimated epidemiological roles in TBP transmission. *Haemaphysalis* spp., such as *Haematopota camicasi* and *Haemaphysalis houyi*, occurred on Nile monitors, monkeys, antelopes, four‐toed hedgehogs, and red‐flanked duikers [42]. This wide spectrum of host diversity, including reptiles, primates, antelopes, and small mammals, suggests potential bridging capacity. Other species, including *Haemaphysalis leachi* and *Haemaphysalis parmata*, exhibited narrower host ranges [42] but were also encountered on livestock carcasses [39, 48], suggesting adaptability or opportunistic infestation. Reports of *Ixodes* spp., including *Ixodes rasus*, *Ixodes aulacodi*, and *Isidus moreli*, are rare and restricted to wildlife in Cameroon and Ghana [38, 42]. The limited reporting likely reflects surveillance bias rather than true absence, as *Ixodes* ticks have been recorded in Africa [74] Similarly, soft ticks (Argasidae) are infrequently encountered at African LWMs. *Argas persicus* infestations on Nigerian poultry markets reached ∼60% on chickens [32] but were lower on turkeys and guinea fowl [75]. Its strong host preference for chickens [32] suggests a significant but overlooked route of zoonotic exposure in poultry systems. Other species, such as *Ornithodoros moubata*, *Argas walkerae*, and *Ornithodoros savignyi*, are less commonly reported [32], and their public health implications in market settings remain under‐explored. These understudied nondominant ticks may contribute to zoonotic risk through host generalism (*Haemaphysalis*), under‐recognized wildlife associations (e.g., *Ixodes*), or neglected poultry‐borne pathways (Argasidae).

### 4.2 Tick‐Borne Diseases of Zoonotic Concern at African LWMs

#### 4.2.1 Rickettsiae

*Rickettsiae* of the spotted fever group (SFGR) emerge as a common zoonotic pathogen group across African LWMs. One notable member of this group is *Rickettsia africa*e, an important pathogen implicated in African tick‑bite fever [76]. High prevalence rates (>75%) of *R. africae* were detected in *Amblyomma* (*A. variegatum*, *A. compressum*, and *A. lepidum*) ticks collected from cattle at LWMs [39, 77]. *A. variegatum* remains the principal vector of *R. africae* [65, 76, 78], but the pathogen has also been detected in other genera, including *Rhipicephalus* and *Hyalomma*. These include ticks such as *R. decoloratus*, *Rhipicephalus appendiculatus*, and *R. annulatus* at LWMs [39, 42, 53, 65]. Possibly, the ecological plasticity within the *Ambylomma* influences the now‐near continent‐wide distribution of ATBV because its first report in 1992 in Zimbabwe [79]. Molecular analyses have revealed three circulating variants of *R. africae* variants I, II, and III [53], highlighting its regional strain diversity [39], which complicates diagnosis and may influence pathogenicity.

Another SFGR species, *Rickettsia aeschlimannii*, was detected in *Hyalomma* ticks collected from cattle, camels, and equids, with *H. rufipes*, *H. impeltatum*, and *H. truncatum* showing the highest infection rates [36, 65, 77, 80]. Zoonosis of this SFGR was first reported in Morocco [81] but was widespread in Africa. Additionally, *Candidatus Rickettsia africaustralis* was identified for the first time in *I. rasus* from pangolins, suggesting either geographic expansion or previously underreported circulation of novel strains [36, 42].

#### 4.2.2 Crimean‐Congo Hemorrhagic Fever Virus (CCHFV)

The CCHFV causes severe hemorrhagic fever in humans [82]. Yet, tick‐borne CCHFV disease transmission is still lacking in many countries [83]. However, seroprevalence studies detect high rates of carcass infection, signaling a significant risk of zoonotic spill‐over. For instance, between 72% and 100% prevalence in camel carcasses was detected in slaughterhouses in Algeria [83]. In their study, Degui et al. [83] identified ticks as the risk factor for CCFV circulation. The virus has been detected in *H. marginatum* (22.4%), *H. dromedarii* (16.4%), and *H. excavatum* (9.6%) infesting cattle carcasses [53], keeping up with reports stating that *Hyalomma* ticks are the principal vectors [84]. However, other *Hyalomma* ticks, including *H. impeltatum*, *H. marmoreum*, *H. truncatum*, or *H. turanicum*, showed 0% infection [53]. Interestingly, the virus has also been detected in *Ambylomma* (*A. lepidum*, *Ambylomma cohaerens*, and *A. variegatum*), *Dermacentor*, and *Rhipicephalus* (including *Rhipicephalus decolaratus*) ticks collected from cattle at LWMs [39, 65, 85]. For instance, genetic studies revealed that the CCFV African genotype II was circulating in Africa and was detected in *R. decoloratus* and other *Rhipicephalus* ticks from cattle [39, 47].

#### 4.2.3 Anaplasmatacae

At African LWMs, seroprevalence studies have identified multiple *Anaplasmataceae* pathogens across diverse host and tick associations. The highest reported infection rate was 55% in camel carcasses sampled in Egypt [86], markedly exceeding the 6.5% and 5.0% infection rates observed in goat and sheep carcasses, respectively, in Nigeria [87]. Species detected include *Anaplasma phagocytophilum*, *Anaplasma platys*, *Anaplasma ovis*, and *Anaplasma bovis*. Zoonotic infections with *A. phagocytophilum*, *A. platys*, and *A. ovis* have been documented in humans [88, 89, 90], and recent evidence suggests zoonotic potential for *A. bovis* as well [91]. *A. ovis* is primarily associated with *Rhipicephalus bursa*, whereas *R. sanguineus* is considered the presumptive vector of *A. platys* [88, 90]. However, both pathogens have also been detected in *R. decoloratus*, *R. appendiculatus*, *R. evertsi*, and other *Rhipicephalus* species collected from cattle and goat carcasses [39]. Grasscutter‐associated *I. aulacodi* tested positive for *Anaplasmataceae*; one for an *Ehrlichia* sp. closely related to strains from *Ixodes ricinus* and *Ichthyococcus ovatus*, and another for an *Anaplasma* sp. with partial similarity to *A. phagocytophilum* [38]. An uncultured *Ehrlichia* sp. was also identified in *H. truncatum* ticks from cattle carcasses in Ghana [65], indicating the circulation of other potentially zoonotic *Anaplasmataceae*.

#### 4.2.4 *Coxiella* and *Candidatus Midichloria mitochondrii*

The detection of *Coxiella burnetii*, the causative agent of Q fever, across multiple livestock–wildlife markets (LWMs) in Africa highlights its complex and poorly characterized epidemiology. In Ethiopia, infection was confirmed in 11.79% of cattle carcasses [92], whereas seroprevalence studies revealed more modest rates of 1.7% in Egyptian camel carcasses [86], 5.9% in Zambian sheep and goats [93], and 9.4%, 4.3%, and 0.9% in South African cattle, sheep, and pigs, respectively, alongside the identification of eight distinct genotypes [40]. The pathogen's detection in *A. variegatum* ticks [65] suggests the widespread presence of *Coxiella‐*like endosymbionts [39]. Adding to this, *H. rufipes* ticks collected from cattle carcasses were found to harbor *Candidatus Midichloria mitochondrii* [65], an endosymbiont whose ecological role is unclear. These findings highlight the diversity of microbial agents present in ticks at LWMs and reinforce the need for expanded surveillance that can differentiate between pathogens and commensals, track genotype distribution, and inform public health. Highly sensitive and specific methods (e.g., tNGS and qPCR) of identifying *C. burnetii* and other endosymbionts should be considered [94].

## 5 Limitations to Our Knowledge of TTBPs at African LWMs

Understanding the true extent of the risk posed by TTBPs risk at African LWMs is hampered by several major constraints: (i) inadequate surveillance and sampling efforts, (ii) underdiagnosis, (iii) geographic bias, (iv) methodological insufficiency, (v) legal and funding constraints for research, and (vi) weak policy integration or enforcement.

First, surveillance efforts of zoonotic diseases in Africa remain inconsistent [6], ad hoc, and weak. Although wildlife wet markets have been linked to pandemics such as SARS‐CoV‐2 [95], equivalent monitoring for tick‐borne zoonoses at LWMs in Africa is lacking. Even when infections occur, they frequently remain undetected. This means cases linked to wildlife exposure can be misclassified or missed entirely. Second, gross geographic bias persists in the regional monitoring of tick and tick‐borne zoonotic risks across African LWMs, corroborating with geographic patchiness in zoonotic monitoring reported in a systematic review by Ateudjieu et al. [6]. This uneven distribution of research creates a skewed understanding of risk. As a result, the Southern regions of Africa appear safer simply because (i) they have not been studied thoroughly and (ii) may lack a vibrant bushmeat trade network.

Third, many key studies (e.g., [32, 48]) rely on opportunistic abattoir sampling that lacks molecular confirmation of tick identity. This limited access to modern equipment and standardized protocols can constrain the depth and reliability of findings, raising questions about under and incorrect identifications. This is particularly true in the case of engorged ticks, which can potentially make up ∼80% of tick collections [65]. Furthermore, funding and strict ethical permitting processes intended to protect further compound these challenges through cost and delay, leaving most research gaps unaddressed. Lastly, even when scientific evidence exists, translating it into effective policy and practice can be difficult.

## 6 Awareness, Perception, and Socio‐Economic Drivers of Knowledge of Tick‐Borne Zoonotic Concerns

Awareness of ticks as vectors of zoonotic disease remains strikingly low across African LWMs but shows significant sociodemographic variation. In North, East, and West Africa, 55%–96.7% of stakeholders are generally uninformed of tick‐borne zoonoses [43, 51, 96, 97, 98]. As a result, 58.9% of stakeholder was unfamiliar with specific tick‐borne, such as CCHFV [99]. Thus, stakeholders are unable to recognize zoonotic from non‐zoonotic pathogens, but perceive imported [48, 53, 100] and domestic animals to be of greatest threat [101]. In contrast, Southern Africa show higher levels (>50%) of knowledge of tick‐borne zooonosis [102, 103]. However, even where awareness is high, knowledge remains heterogenous across provinces [34] and between urban (66.7%) and rural (17.2%) areas [104]. As a result, awareness of specific zoonotic diseases is also low in rural spaces.

Occupational roles show distinct differences in awareness of tick‐borne zoonosis along Africa's meat value chain. For example, ∼88% of hunters are more likely than traders (∼64%) to understand the basic causes of zoonotic infections [105]. Possibly, knowledge passed from generation to generation, and hands‐on exposure reinforces a mental link between animal pathogens and human illness. The latter seems unlikely since traders are more likely to butcher animals exposing themselves to animal fluids and zoonotic diseases [52]. Hunters (∼65%) were less clear on the role of public health services compared to traders (∼38.1%) [51], possibly because hunters operate in rural settings, with limited access to veterinary or public health outreach. Together, these findings suggest that occupational exposure shapes awareness in uneven ways. However, attitudes towards zoonotic diseases remain uniform between the groups. For instance, both groups deem protective equipment unnecessary [51].

These knowledge gaps are shaped by socio‐economic and demographic drivers that vary between communities and occupations. For instance, deep‐rooted cultural beliefs [102] that wild meat is pure and incapable of harboring pathogens [52], combined with low levels of formal education [35], create a false sense of safety and ignorance of zoonotic risks. However, age appears protective along the meat value chain [103], and even among veterinary students in North Africa [106]. Possibly, learned experiences play a role in mitigating cultural misconceptions.

## 7 TTBP Control Along the Wild Meat Value Chain

The threat of tick infestation and TBPs extends from livestock producers and wildlife hunters, butchers, and market vendors to the end consumers in African livestock–wildlife markets (LWMs).

### 7.1 Livestock Farmers and Wildlife Hunters

Tick control strategies in African livestock systems are nearly universal across Africa, with over ∼75% of farmers in Africa reliant on acaricides [98, 103, 107]. This widespread adoption is shaped by financial capacity and cultural preferences [34]. The alternative is to use ethno‐veterinary and household remedies [102], such as aloe, ash, or cigarette residue, which are preferred due to rapid action and blood‐repellent effects [34]. Indeed, ethno‐veterinary plants have shown arthropod toxicity, antifeedant, anti‐ovipositant, and repellent properties [108], suggesting potential of diverse and natural anti‐tick agents. However, recent evidence shows that most farmers believe in the efficacy of chemical acaricides [98, 107]. Other farmers manually remove ticks and crush them [98]; adjust dipping frequency by season ([34, 107]; select tick‐resistant breeds [103]; or prefer animals with lighter coat color and short hairs [34, 102]. Most likely, African hunters conduct regular tick checks following each hunt and use local plants for treatment [109]. However, such information is highly lacking in Africa. Evidence elsewhere suggests that protective behaviors amongst hunters include the use of skin and clothing repellents [110, 111].

### 7.2 Slaughterhouses and Markets

Hygienic behaviors (e.g., hand sanitizing) and wearing personal protective equipment (PPE) (e.g., gloves) are recognized as effective measures to prevent food‐borne infections at wet meat markets [112, 113]. However, many African LWM actors overlook essential sanitary measures, even in contexts where individuals recognize the dangers of exposure to animal fluids [61]. For instance, a high proportion of hunters (96%), informal butchers (98%) [114], and slaughterhouse workers reportedly do not use protective clothing. Most people wipe hands with anything from newspapers to grass [115] or wash hands with beer or palm wine after butchering [52]. Reluctance to manage the risk of zoonoses could be due to attempts to avoid raising concerns about product safety [52] or a genuine lack of PPE provisions [61]. Nevertheless, such practices violate food safety and handling standards [116] and expose consumers to possible TBPs. These findings highlight two critical issues: (a) That risk knowledge does not always translate into protective practices and (b) inadequate regulatory interventions.

Rudimentary meat preservation methods are widespread across African LWMs, underscoring the lack of investment in public health infrastructure [25]. For instance, internal organs (e.g., intestines) are removed from carcasses to delay spoilage and extend shelf life [52]. Additionally, meat is cooked [61]. However, ambient tropical temperatures can accelerate pathogen proliferation [117], and the mixing of species and body parts during transport and storage creates opportunities for TTBP cross‐contamination [52]. Together, these findings suggest that improving hygiene at wet meat markets is not just a matter of education or awareness. It requires investment in public health infrastructure, economic empowerment, and policy support [25].

## 8 Conclusion and Future Directions

African LWMs are unique epidemiologically permeable hubs where livestock, wildlife, humans, and vectors intersect. Limited surveillance evidence confirms *Amblyomma*, *Rhipicephalus*, and *Hyalomma* ticks as widespread vectors of pathogens, including *R. africae*, *Anaplasmataceae*, CCHFV, and *C. burnetii*. Yet, there is limited knowledge of tick and tick‐borne zoonotic risk at LWMs, overreliance on wildlife trade and inconsistent access to protective equipment. Together, these factors magnify the risk of tick‐borne zoonosis at LWMs markets. As a result, the spillover risk at LWMs is not solely a biological phenomenon but also a social and structural one. Addressing this interconnected risk requires integrated One Health responses that transcend surveillance and embrace prevention, innovation, and community partnership. Effective strategies should combine ecological monitoring with locally feasible interventions, such as developing ethno‐veterinary acaricides that are culturally acceptable and economically sustainable. Focus should be directed towards (i) longitudinal surveillance efforts using sensitive and specific monitoring methods, (ii) education campaigns tailored for local contexts, and (iii) creating multidisciplinary One Health task forces to promote the routine use of protective gear and hygienic practices and strengthen enforcement of market health and safety standards.

## Author Contributions

**Allen Takudzwa Munaro**: investigation, writing and editing of the original draft.

## Ethics Statement

The author has nothing to report.

## Conflicts of Interest

The author declares no conflicts of interest.

## Data Availability

Data sharing is not applicable as no original data was used.
